# Bioinformatics identification of copyback and multihost-adapted defective viral genomes in dengue virus

**DOI:** 10.3389/fcimb.2026.1825608

**Published:** 2026-05-20

**Authors:** Jianhai Yu, Hao Wu, Yan Zhan, Yuan Liang, Linlin Xiang, Xuling Liu, Xiaoting Xie, Li Zhu, Qinghua Wu, Weiwei Xiao, Chengsong Wan, Chenguang Shen, Bao Zhang, Wei Zhao

**Affiliations:** 1 BSL-3 Laboratory (Guangdong), Guangdong Provincial Key Laboratory of Tropical Disease Research, Key Laboratory of Infectious Diseases Research in South China, School of Public Health, Southern Medical University, Guangzhou, Guangdong, China; 2 Zhuhai Center for Disease Control and Prevention, Zhuhai, Guangdong, China; 3 KU Leuven Department of Microbiology, Immunology and Transplantation, Rega Institute for Medical Research, Virology, Antiviral Drug & Vaccine Research Group, Leuven, Belgium

**Keywords:** copyback DVGs, defective viral genomes, dengue virus, multihost-adapted DVGs, next-generation sequencing

## Abstract

**Introduction:**

Defective viral genomes (DVGs) have been detected in clinical samples, and their antiviral effects have been verified. However, due to the limitations of traditional methods, all the reported DENV DVGs to date are Deletion DVGs, and it has not been proven that these DVGs can be stabilized across different hosts.

**Methods:**

We used the bioinformatics software DVGfinder with various dengue virus NGS data, including samples from patients, *Aedes albopictus*, C6/36 cells, and Vero cells, to identify DVGs. We compared the distribution of DVGs across different hosts and analyzed the dynamics of DENV-1 DVGs during serial passaging, identifying DVGs that are stably maintained across hosts.

**Results:**

First of all, we found copyback type DVGs in different datasets, with the dominant DVGs in patient sera being 3’ copyback. Secondly, we observed that in C6/36 cells and *Aedes albopictus*, the DVGs did not change significantly with DENV-1 passages, while in Vero cells, the number of specific Deletion DVGs continuously increased with DENV-1 passage. Finally, using clustering algorithms, we identified a set of candidate deletion DVGs predicted to stably exist across different hosts. One of these candidates, designated DeletionA6 (BP 1320, RI 7700), was experimentally validated by nested PCR in sera from patients infected with DENV-1 to DENV-4.

**Discussion:**

This study describes the distribution patterns of DVGs across different samples and provides preliminary bioinformatic evidence for a subset of deletion DVGs.

## 1 Introduction

Defective virus genomes (DVGs) are viral replication by-products produced when the RNA-dependent RNA polymerase (RdRp) is detached from the replication template and binds to the template again (Ziegler and Botten, 2020). The site at which the RdRp detaches from the template was defined as the breakpoint (BP), and the site at which the RdRp rebinds to the template again was defined as the reinitiation (RI). Depending on the generation mechanism, DVGs can be classified into two main types: copyback and deletion. Copyback DVGs contain the intact 5’-end or 3’-end of the viral genome, as well as complementary sequences to form a stem-loop structure. Deletion DVGs contain partially missing genes (Vignuzzi and López, 2019).

DVG assembles into defective interfering particles (DIPs) upon binding to structural proteins. DVG was first identified during the continuous passage of influenza viruses with a high multiplicity of infection (MOI) (Von Magnus and Gard, 1947), and has subsequently appeared in almost all virus families (Alnaji and Brooke, 2020; Pitchai et al., 2024). Initially, the presence of DIPs was verified by detecting viruses with high MOI in successive passages using plaque formation experiments (Ziegler et al., 2016). Subsequently, with the development of PCR technology, DVGs could be detected using specific primers (Calain et al., 1992). Currently, high-throughput sequencing technology is applied to detect DVG, and detection software, such as ViReMa-a (Routh and Johnson, 2014), DI-tector (Beauclair et al., 2018), VODKA (Sun et al., 2019), and DVG-profiler (Bosma et al., 2019), has been developed, which can detect all DVGs in samples and describe their abundance. To improve the sensitivity and specificity of the software, DVGfinder (Olmo-Uceda et al., 2022) integrates two commonly used DVG detection software packages, ViReMa-a and DI-tector, and adds a machine-learning module to evaluate the true presence of the jointly detected DVG. To facilitate DVG description, DVGfinder uses BP and RI to describe the detected DVG.

DVGs can inhibit the replication of the parental virus by competing for viral resources and activating the innate immune pathway. Owing to its short length, DVG has a significant replication advantage, and the proportion of DVG increases significantly as viral replication proceeds. DVG can compete with the parental virus for viral resources, such as viral replicative enzymes and structural proteins, thus reducing the resources available to the parental virus to complete the viral cycle and inhibit replication of the parental virus (White et al., 1998; Xie et al., 2017). DVGs can activate the innate immune pathway by virtue of their sequence characteristics, especially copyback DVG, which is one of the most potent immune agonists known to date.

Over the past two decades, the global incidence of dengue has increased dramatically, with reported cases surging ten-fold from 2000 to 2019. Following a historic peak in 2019, transmission has remained intense. Since the beginning of 2023, an unexpected spike has led to a near-record high of over five million cases and more than 5, 000 deaths reported across over 80 countries. However, the true global burden is likely underestimated, as most primary infections are asymptomatic and reporting is not mandatory in many countries (World Health Organization, 2023). The ability of dengue viruses to cause antibody-dependent enhancement has hindered the development of vaccines and neutralizing antibodies, and the risk of resistance to conventional broad-spectrum antivirals has created an urgent need for novel antiviral and control tools (Kok et al., 2023). DIPs, which specifically inhibit viral replication and can adapt to viral mutations, are a promising novel antiviral strategy that has already been applied in antiviral studies on ZIKV (Rezelj et al., 2021) and SARS-CoV-2 (Zhou et al., 2022).

In this study, we used DVGfinder in conjunction with DENV short-read NGS data for DENV DVGs. First, we characterized DVGs in clinical and non-clinical samples and subsequently identified multihost-adapted DVGs as antiviral candidates by integrating the characterization of DVGs in all samples. Finally, we characterized DVGs produced by DENV-1 during successive passages in different hosts.

## 2 Materials and methods

### 2.1 Software and NGS data

In this study, we use the Bioproject database in NCBI (www.ncbi.nlm.nih.gov) to search the NGS data available, using the retrieval terms “DENV” or “Dengue Virus” for studies up to January 31, 2023 (**Supplementary Table 1**).

To compare DVG abundance across different samples (clinical and non-clinical), the total number of detected DVGs in each sample was normalized to 1, 000, 000 reads. Abundance of each DVG type (e.g., 3’ copyback, 5’ copyback, and deletion) was then expressed as the proportion of that DVG type relative to the total DVGs within the same sample.

We used the bioinformatics software DVGfinder (GitHub - MJmaolu/DVGfinder: Defective Viral Genome finder). Environment setup and software installation are in accordance with the User’s Guide. The NGS data and corresponding reference DENV which were required for the initiation of DVGfinder, were shown in **Supplementary Table 1**. Adapter sequences were trimmed using Trimmomatic software. The quality of the high-throughput sequencing data was assessed using FastQC software, and reads with a quality score above 30 were retained for downstream analysis. DVGfinder can output results, which contain the characteristics of DVG.

The most important of these results are the BP and RI, and R (version 4.4.0) were used to cluster the detected Deletion DVGs to find similar Deletions that can coexist in different hosts according to DVGs characteristics. For DBSCAN (Density-Based Spatial Clustering of Applications with Noise) clustering, the parameters (epsilon and minPts) were set according to the distribution characteristics of the BP and RI sites. Detailed parameter values, DBSCAN methods, and the datasets used for clustering are provided in the **Supplementary Materials** (**Supplementary Table 1**, **S2** and Methods S2.1) (Ester et al., 1999). A double-sided P < 0.05 was regarded as statistically significant. All statistical analysis was performed by the R software (version 4.4.0) and SPSS 23.0 (IBM SPSS Statistics, Armonk, NY, USA).

### 2.2 Cell lines and virus preparation

C6/36 cells, a mosquito cell line from *Aedes albopictus*, were grown in RPMI-1640 supplemented with 10% FBS at 28 °C. Vero cells, an African green monkey kidney cell line, were grown in DMEM supplemented with 5% FBS at 37 °C. DENV-1 (GenBank: NC_001477.1) was grown in C6/36 cells for 2–3 days, and in Vero cells for a week.

### 2.3 Nested PCR for verification of host-adapted DVG

We used 10^*^7 plaque-forming units (PFU) of DENV-1 to infect C6/36 as well as Vero cells for five consecutive passages with 0.1 multiplicity of infection (MOI) and then collected C6/36 as well as Vero-derived DENV-1 with titers of 5 × 10*6 PFU as well as 6 × 10*6 PFU, respectively. DENV-1 cell culture and DENV1–4 patient serum were collected and then extracted viral nucleic acids using the NucleoSpin^®^ RNA Virus mini kit (Takara Co., Ltd), and reverse transcription was performed using the Evo M-MLV RT Premix for quantitative polymerase chain reaction (Accurate Bio-technology Co, Ltd). Nested PCR was performed using Easy-Load™ PCR Master Mix (green, 2X) from Beyotime Co, Ltd. The deletion A6 outer primer was F: TTTTGGGACTGCGTAT, outer primer R: TGACTTCTTGCCTTGTT; deletion A6 inner primer was F: ACCACCTTCATCATCG and inner primer R: TGACTTCTTGCCTTGTT. All nested PCR assays were performed under stringent conditions to minimize contamination risk, including physical separation of pre-PCR and post-PCR areas, use of dedicated reagents and filter pipette tips, and inclusion of no-template controls (NTC) and uninfected cell extraction blanks in each reaction set. A representative gel image showing the negative control lanes (no bands) is provided in **Supplementary Figure 5**.

### 2.4 Multihost-adapted DENV DVGs

DVGs are by-products of viral replication, and DENV DVGs have been detected in both *Aedes albopictus* and patients. DVGs inhibit viral replication not only in *Aedes albopictus* but also in patients, effectively protecting them, and have great potential to become a new type of antiviral. However, thousands of DVGs are produced along with viral replication, and we need to find DVGs that can be stabilized and exist in different hosts. Therefore, it is crucial to screen DVGs that can be stabilized in different hosts, which we define as multihost-adapted DVGs. These need to meet the following conditions: (1) be consistently detected across all serial passages of dengue virus in different susceptible cells as well as in *Aedes albopictus*; and (2) be able to be detected in patient serum.

It is important to clarify that the term “multihost-adapted” is used operationally in this study to describe DVGs that are consistently detectable across different host-derived samples. This terminology does not imply confirmed cross-host transmission, independent packaging, or evolutionary adaptation of these DVGs. Their consistent detection across hosts is likely associated with intrinsic sequence or structural features of the viral genome. The term “adapted” is therefore used descriptively rather than mechanistically.

### 2.5 Clinical sample collection and ethics statement

Serum samples from 10 patients infected with DENV-1 to DENV-4 were obtained from the Guangzhou Eighth People’s Hospital. These were residual diagnostic specimens from routine clinical testing of suspected dengue patients and were not prospectively collected for this study. The retrospective use of these de-identified residual samples for research purposes was approved by the Ethics Committee of the Guangzhou Eighth People’s Hospital. Broad informed consent for future research use had been obtained from patients at the time of initial clinical sampling, and the Ethics Committee waived the requirement for re-consent for this specific retrospective analysis. The processing of serum samples and the detection of defective viral genomes were conducted at Southern Medical University. This study does not involve any epidemiological or personal information related to patient privacy; all personal identifiers were removed prior to transfer to the researchers, and only the DENV serotype was disclosed. The detection of clinical samples and analysis of DVGs have no impact on patient diagnosis or treatment.

## 3 Results

### 3.1 Copyback DVGs are the predominant DVG type in clinical samples

To facilitate comparison of DVG abundance between clinical and non-clinical samples, the total number of detected DVGs in each sample was normalized to 1, 000, 000.

**Figure 1:**
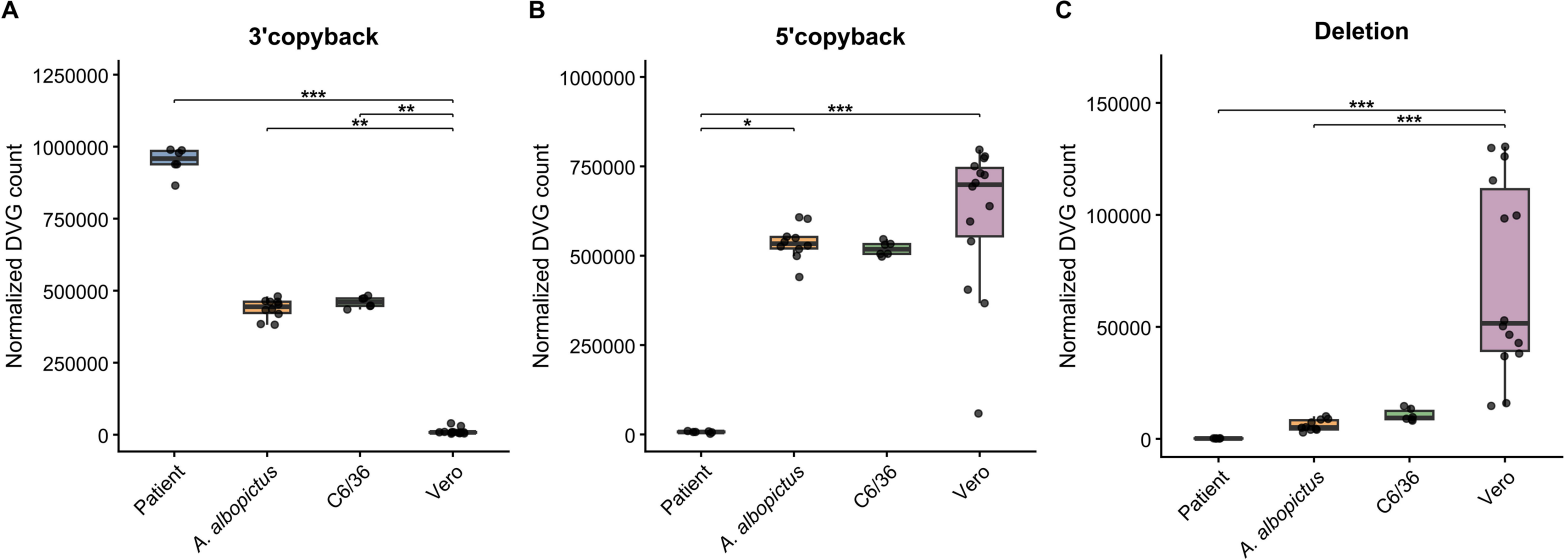
3’ copyback DVGs, 5’ copyback DVGs and Deletion DVGs are present in human clinical samples. **(A–C)** DVG counts, unique junctions normalized to 1,000,000 reads. The Kruskal-Wallis test was used to compare DVG abundance across different hosts, followed by Dunn’s multiple comparisons test for post hoc analysis. * indicates P<0.05, **indicates P<0.01, ***indicates P<0.001. **(A)** 3’ copyback DVGs: The abundance in clinical samples was significantly higher than in Vero cells (p < 0.001); the abundance in C6/36 cells and *Aedes albopictus* was also significantly higher than in Vero cells (p < 0.01). **(B)** 5’ copyback DVGs: The abundance in clinical samples was significantly lower than in Vero cells (p < 0.001); the abundance in *Aedes albopictus* was significantly higher than in clinical samples (p < 0.05). **(C)** Deletion DVGs: The abundance in Vero cells was significantly higher than in clinical samples and *Aedes albopictus* (p < 0.001).

Next, we compared the abundance of 3’ copyback, 5’ copyback, and deletion DVGs across different hosts using the Kruskal-Wallis test, followed by Dunn’s multiple comparisons test (**Figure 1**). Significant host-dependent differences were observed for all three DVG types. For 3’ copyback DVGs, abundance was significantly higher in clinical samples than in Vero cells (p < 0.001), and also significantly higher in C6/36 and *Aedes albopictus* compared to Vero cells (p < 0.01 for both). In contrast, 5’ copyback DVGs showed significantly lower abundance in clinical samples compared to Vero cells (p < 0.001) and significantly higher abundance in *Aedes albopictus* compared to clinical samples (p < 0.05). However, no significant differences were observed between Vero cells and either C6/36 or *Aedes albopictus* (p = 0.40 and p = 0.79, respectively), nor between *Aedes albopictus* and C6/36 cells. Deletion DVGs were significantly more abundant in Vero cells than in clinical samples and *Aedes albopictus* (p < 0.001), whereas no significant differences were observed between Vero cells and C6/36 cells or among the other host comparisons.

**Figure 2:**
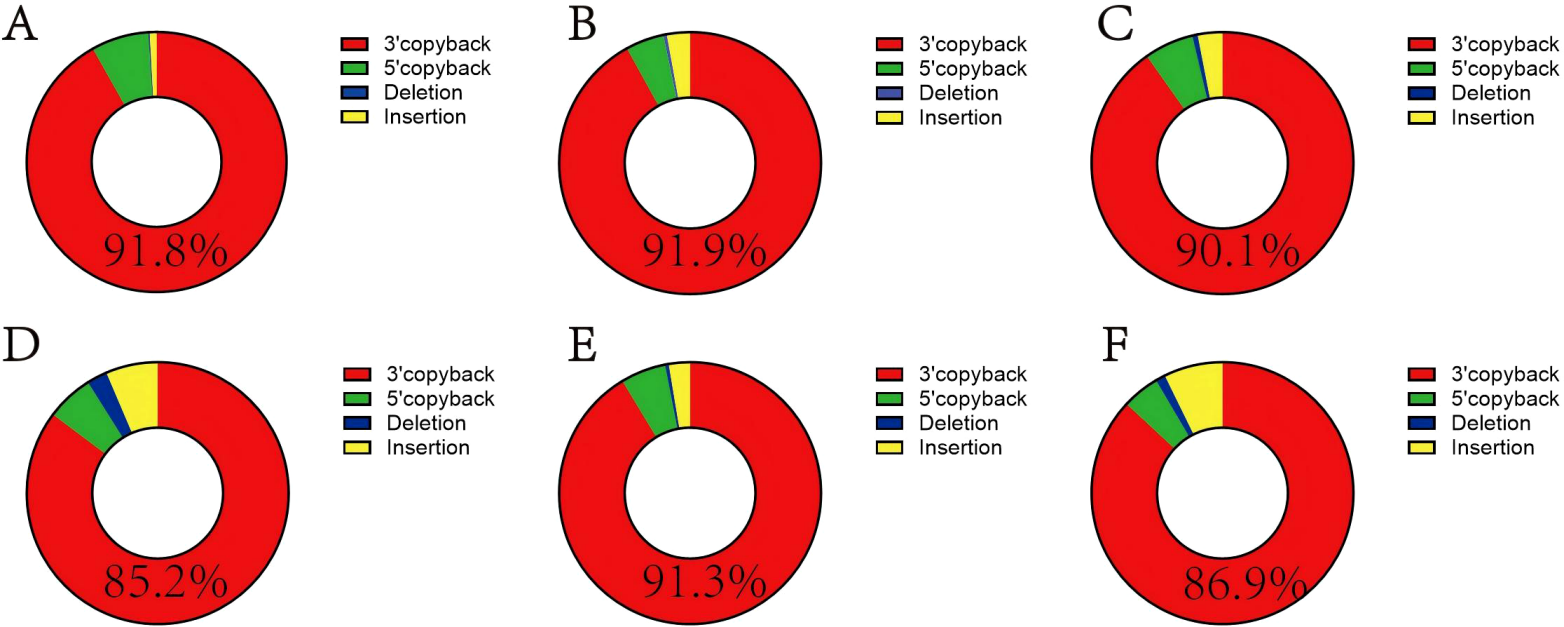
3’ copyback DVG is predominant in patients’ serum. **(A–F)** correspond to patients 1–6, respectively. The proportions of 3’ copyback DVGs were: **(A)** 91.8%, (B) 91.9%, **(C)** 90.1%, **(D)** 85.2%, **(E)** 91.3%, **(F)** 86.9%.

**Figure 3:**
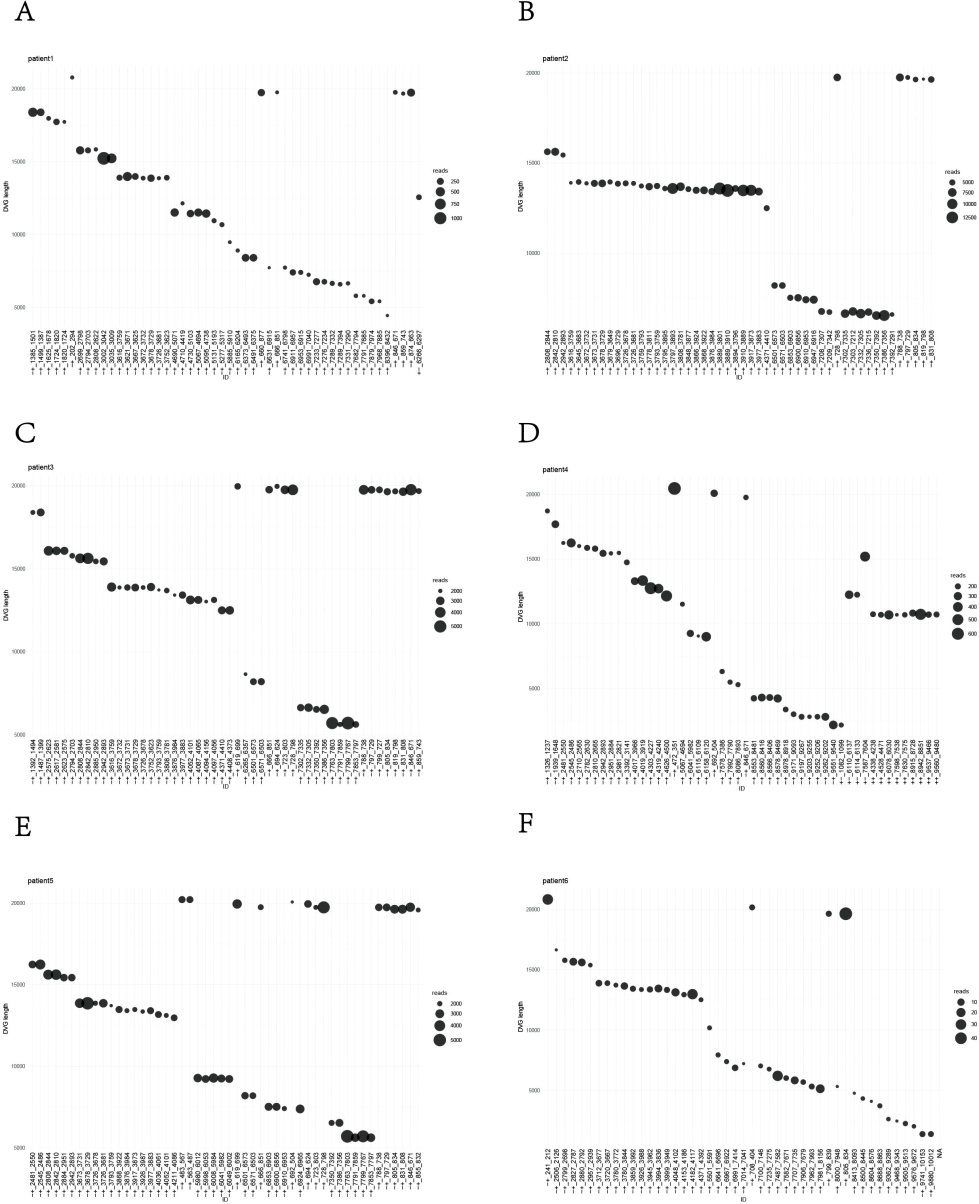
The top 50 most abundant defective viral genomes (DVGs) identified in the sera of six patients are shown. **(A–F)** Data analysis representing 6 patients respectively. The numbers on the x-axis indicate the breakpoint (BP) and reinitiation (RI) sites of each DVG, while the “+” and “–” symbols denote the strand orientation. The y-axis represents the length of the DVGs, and the bubble size corresponds to their abundance. All displayed DVGs are 3’ copyback-type defective viral genomes (with “+-” or “-+” indicating the copyback configuration).

To further explore the abundance characteristics of DVGs in the DENV-1 clinical samples, we counted the proportions of the four DVGs in the six patients and found that the proportion of 3’ copyback was more than 85% in all six patients (**Figure 2**). We ranked the DVGs in descending order of abundance, and the top 50 DVGs with the highest abundance in each of the six patients were all 3’ copyback DVGs, demonstrating that 3’ copyback DVGs predominate in patient sera (**Figure 3**). It should be noted that these findings are based on six clinical samples, and the sample size is limited. The predominance of 3’ copyback DVGs in patient sera, while notable, should be interpreted with caution and requires validation in larger clinical cohorts.

### 3.2 Dynamics of DVGs during DENV-1 serial passage

Compared with the normal genome, DVGs have a faster replication rate and shorter length. Therefore, during viral passage, DVGs are able to continuously self-enhance and increase in abundance. DVGs are produced as a result of virus-host interactions. Therefore, we preliminarily explored the characterization of DVGs produced by sustained passage of DENV-1 in different hosts.

Firstly, we compared the changes in the percentage of different types of DVGs between different DENV-1 (30A and 1806) during passage in different hosts (*Aedes albopictus* and C6/36), and detected the percentage of different DVGs at 1, 5, and 10 passages, and performed multivariate repeated-measures analysis of variance to analyze the effects of different factors on the percentage of different types of DVGs.

**Figure 4:**
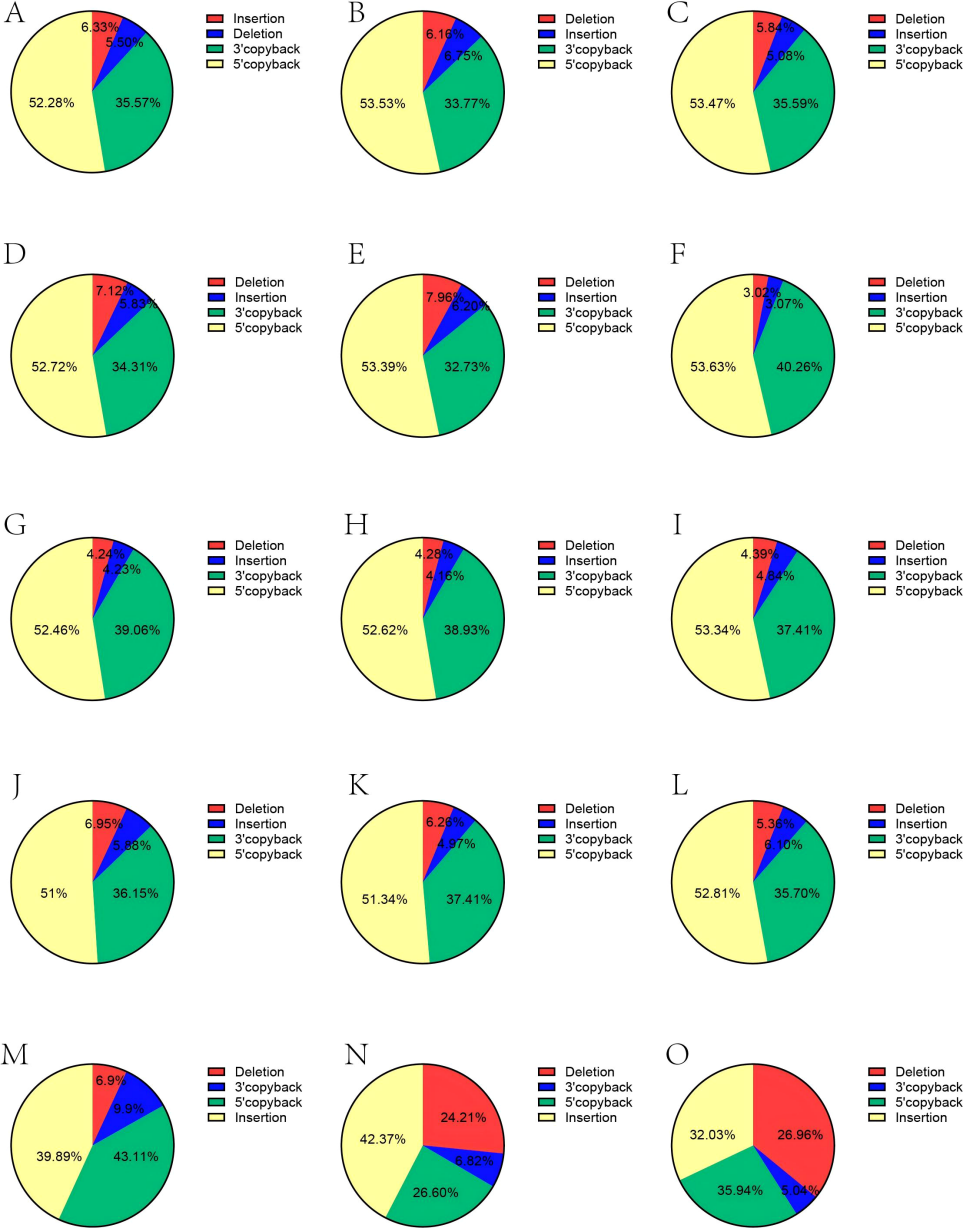
Changes in the proportions of the four different types of DVGs produced by different DENV1s (30A and 1806) during passage in different hosts (C6/36, *Aedes albopictus*, Vero). **(A–C)**. proportion of four types of DVGs at the 1st, 5th and 10th passages of 30A virus in C6/36. **(D–F)**. proportion of four types of DVGs at the 1st, 5th and 10th passages of 30A virus in *Aedes albopictus*. **(G–I)**. proportion of four types of DVGs at the 1st, 5th and 10th passages of 1806 virus in C6/36. **(J–L)**. proportion of four types of DVGs at the 1st, 5th and 10th passages of 1806 virus in *Aedes albopictus*. **(M–O)**. proportion of four types of DVGs at the 1st, 3rd and 5th passages of Thailand 2005 in Vero. Differences in DVG proportions across passages were evaluated using a Chi-square test (p < 0.001).

DENV-1 strains (30A and 1806) were found to have no effect on the occupancy ratio between the four different types of DVGs (P = 0.41); different hosts (C6/36 and *Aedes albopictus*) had no effect on the occupancy ratio between the four different types of DVGs (P = 0.38); the number of passages had no effect on the occupancy ratio between the four different types of DVGs (P = 0.46); There was a significant difference in the percentage between the different types of DVGs (P < 0.001). Therefore, we conclude that the percentage of DVGs produced by DENV-1 in (C6/36 and *Aedes albopictus*) does not vary with the number of passages, DENV-1 type, and host species, and remains relatively stable. Interestingly, the percentage of Deletion DVGs is much smaller than that of Copyback DVGs, while the sum of 5’ copyback DVGs and 3’ copyback DVGs is stable at more than 85% (**Figures 4 A–L**).

We then explored the changes in different types of DVGs with DENV-1 passage in Vero. We found that the proportion of the four types of DVGs changed significantly with the passage of DENV-1 (**Figures 4M–O**), Chi-square test p-value < 0.001. The proportion of Deletion DVGs increased from 9.9% in the first passage to 35.94% in the fifth passage; the proportion of Insertion DVGs declined from 43.11% to 32.03% in the fifth passage; 5’ copyback DVGs decreased from 39.98% in the first pass to 26.96% in the fifth passage; and 3’ copyback DVGs decreased from 6.9% in the first pass to 5.04% in the fifth pass. After that, we focused on Deletion DVGs to explore the reason why the proportion of Deletion DVGs kept increasing with the passages, and we found that a certain class of Deletion DVGs kept accumulating with the passages of DENV-1, and the BP of this class of Deletion DVGs was roughly between 0-1000, and the RI was roughly between 2000-3000 (**Supplementary Figure 1**).

Next, we initially explored the highest abundance copyback DVGs in each passage of DENV-1 (1806 and 30A) in *Aedes albopictus* and C6/36, and we plotted bubble plots based on BP, RI, and abundance information to investigate the distribution of high abundance copyback DVGs. The distribution of 3’ copyback DVGs is shown in **Supplementary Figure 2**, and the distribution of 5’ copyback DVGs is shown in **Supplementary Figure 3**. We found that the highest abundance DVGs do not change with the times of passages, nor with the host, and that the same DENV-1 produces the same highest abundance copyback DVGs in *Aedes albopictus* and C6/36. However, different DENV-1s do not produce the identical highest abundance copyback DVGs in *Aedes albopictus* and C6/36 cells, and we summarize the highest abundance copyback DVGs produced by different DENV-1s in *Aedes albopictus* and C6/36 in **Table 1**. Interestingly, both 30A and 1806 were able to produce the same high abundance of 5’ copyback DVGs as well as 3’ copyback DVGs.

**Table 1:** The highest abundance of copyback DVGs produced by DENV1.

DENV1	Host	5’ copyback DVGs	3’ copyback DVGs
30A	C6/36	+-_4095_4041 +-_1997_1868	-+_9328_9364 -+_7641_7676 -+_10412-10432
30A	*Aedes albopictus*	+-_4095_4041 +-_1997_1868	-+_9328_9364 -+_7641_7676 -+_10412-10432
1806	C6/36	+-_4095_4041 +-_417-361	-+_8116_8347 -+_9328_9364 -+_7641_7676
1806	*Aedes albopictus*	+-_4095_4041 +-_417-361	-+_8116_8347 -+_9328_9364 -+_7641_7676

For 5’ copyback DVGs, “+-” indicates the RdRp detaches from the positive-sense strand and reinitiates on the negative-sense strand; for 3’ copyback DVGs, “-+” indicates the RdRp detaches from the negative-sense strand and reinitiates on the positive-sense strand. The numbers represent the genomic positions of the breakpoint and reinitiation site on the DENV-1 reference genome (GenBank: NC_001477.1).

### 3.3 Identification of multihost-adapted DENV deletion DVGs

**Figure 5:**
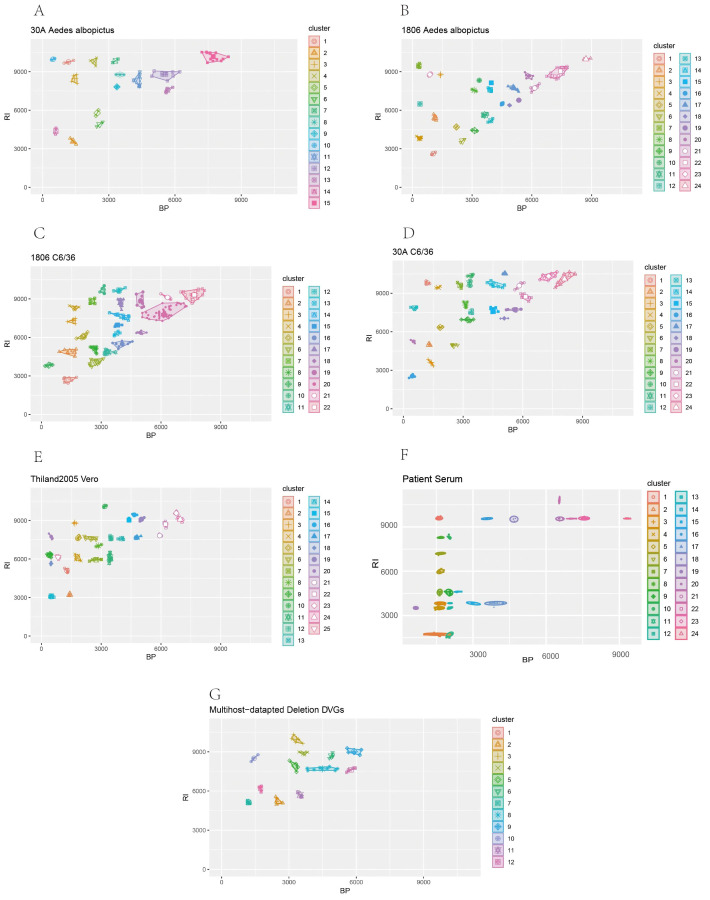
Prediction of dengue virus multihost-adapted deletion DVGs. We first screen out host-adapted DVGs with different dengue viruses in different hosts. **(A)** it was shown the stable existence of DVGs produced by 30A in *Aedes albopictus*. **(B)** it was shown the stable existence of DVGs produced by 1806 in *Aedes albopictus*. **(C)** it was shown the stable existence of DVGs produced by 30A in C6/36. **(D)** it was shown stable existence of DVGs produced by 1806 in C6/36. **(E)** it was shown stable existence of DVGs produced by Thailand 2005 in Vero. **(F)** deletion DVGs which was detected in DENV3 clinical samples. **(G)** we used DBSCAN clustering method to comprehensively analyze different host-adapted DVGs and DVGs in clinical samples, thus 12 multihost-adapted DVGs were predicted.

In order to find the multihost-adapted DVGs, we first screened the Deletion DVGs that could be continuously detected with the continuous passage of DENV-1 in C6/36, Vero, and *Aedes albopictus*, respectively, and we found that some of these DVGs that were continuously detected had very similar characteristics, and we could not treat these DVGs as different kinds of DVGs. So, we used R language to classify the dissimilar stable-existing DVGs based on their BP as well as RI features. Finally, we pooled the classified Deletion DVGs stably present in different hosts and the DVGs detected in patient sera and analyzed them again using the DBSCAN (Density-Based Spatial Clustering of Applications with Noise) clustering method to filter out the clusters containing patient Deletion DVGs and the stably present DVGs in all the hosts, which are multihost-adapted DVGs. We found 12 host-adapted DVGs (**Figure 5G**). The breakpoint (BP) and reinitiation (RI) sites of these DVGs are shown in **Table 2**.

**Table 2:** Detailed information of the 12 predicted multi-hosted DVGs.

Name	BP	RI
Deletion A1	1253	3811
Deletion A2	2411	5555
Deletion A3	3420	9981
DeletionA4	3683	8761
DeletionA5	3399	7826
DeletionA6	1317	7776
DeletionA7	1266	5069
DeletionA8	4378	7782
DeletionA9	5592	9273
DeletionA10	1775	6366
DeletionA11	4948	8644
DeletionA12	3570	5565

To verify whether our predicted multihost-adapted DVGs are real, we used PCR on different DENV-1 samples, and in this study, we did not choose to clone the full length of the Deletion DVG completely but instead chose to detect its characteristic fragments. According to the principle of Deletion DVG production, there are obvious traces of viral gene splicing on Deletion DVG, so according to our predicted sequence of Deletion DVG, the 5’ primers for nested PCR were designed upstream of the BP of the DVG and the 3’ Primer for nested PCR were designed downstream of RI, and the length of the predicted PCR products was in the range of 500 bp-1 kb.

**Figure 6:**
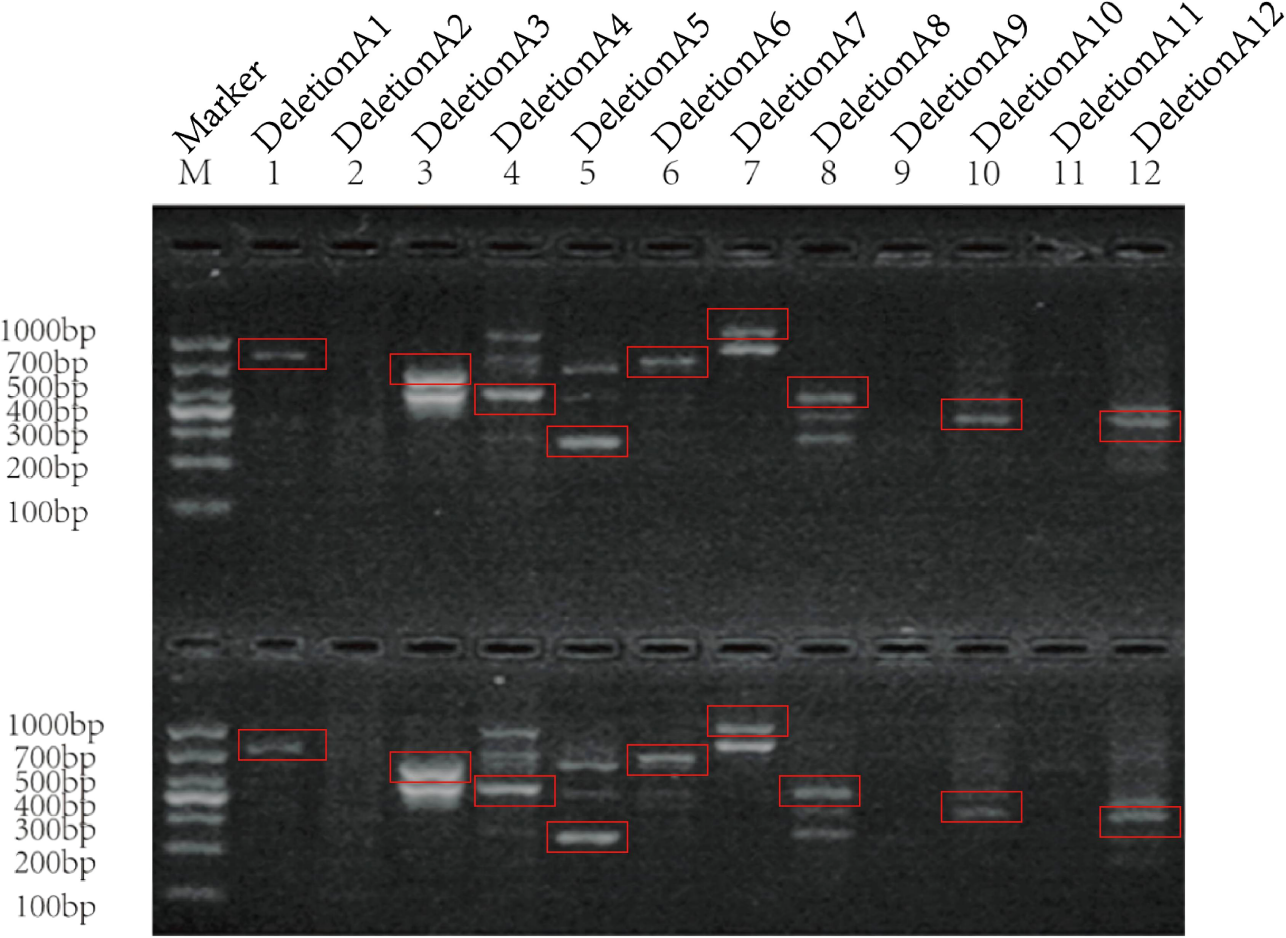
DeletionA6 can be detected in Vero and C6/36 infected by DENV1. Where M is the 1 kd DNA marker, PCR was performed from lane 1 to lane 12 using primers targeting DeletionA1-DeletionA12, respectively. The top half of the agarose gel was used to detect the DVGs in C6/36-derived DENV1, and the bottom half was used to detect the DVGs in the Vero-derived DENV1. Expected bands appeared in the upper and lower parts of lanes 1, 3, 4, 5, 6, 7, 8, 10, and 12.

We first verified whether DeletionA1-A12 was real in DENV-1 cell cultures. We used C6/36 and Vero cells to infect DENV-1 and collected the corresponding DENV-1, then we extracted the viral RNA, and reverse transcribed it into cDNA, then performed nested PCR using specially designed primers and subjected the PCR products to agarose gel electrophoresis, which showed that multiple Deletion DVGs were detected (**Figure 6**). Bands corresponding to the expected sizes were excised from the gel, cloned into plasmid vectors by TA cloning, and further validated by Sanger sequencing. Specifically, bands representing DeletionA1 (lane 1, **Figure 6**), DeletionA6 (lane 6, **Figure 6**), and DeletionA12 (lane 12, **Figure 6**) were successfully recovered and sequenced. Alignment of these amplicons with the DENV-1 reference genome revealed distinct gene-jump junctions characteristic of Deletion DVGs. The BP and RI sites were as follows: DeletionA1 (BP 3100, RI 10029), DeletionA6 (BP 1320, RI 7700), and DeletionA12 (BP 320, RI 3520).

**Figure 7:**
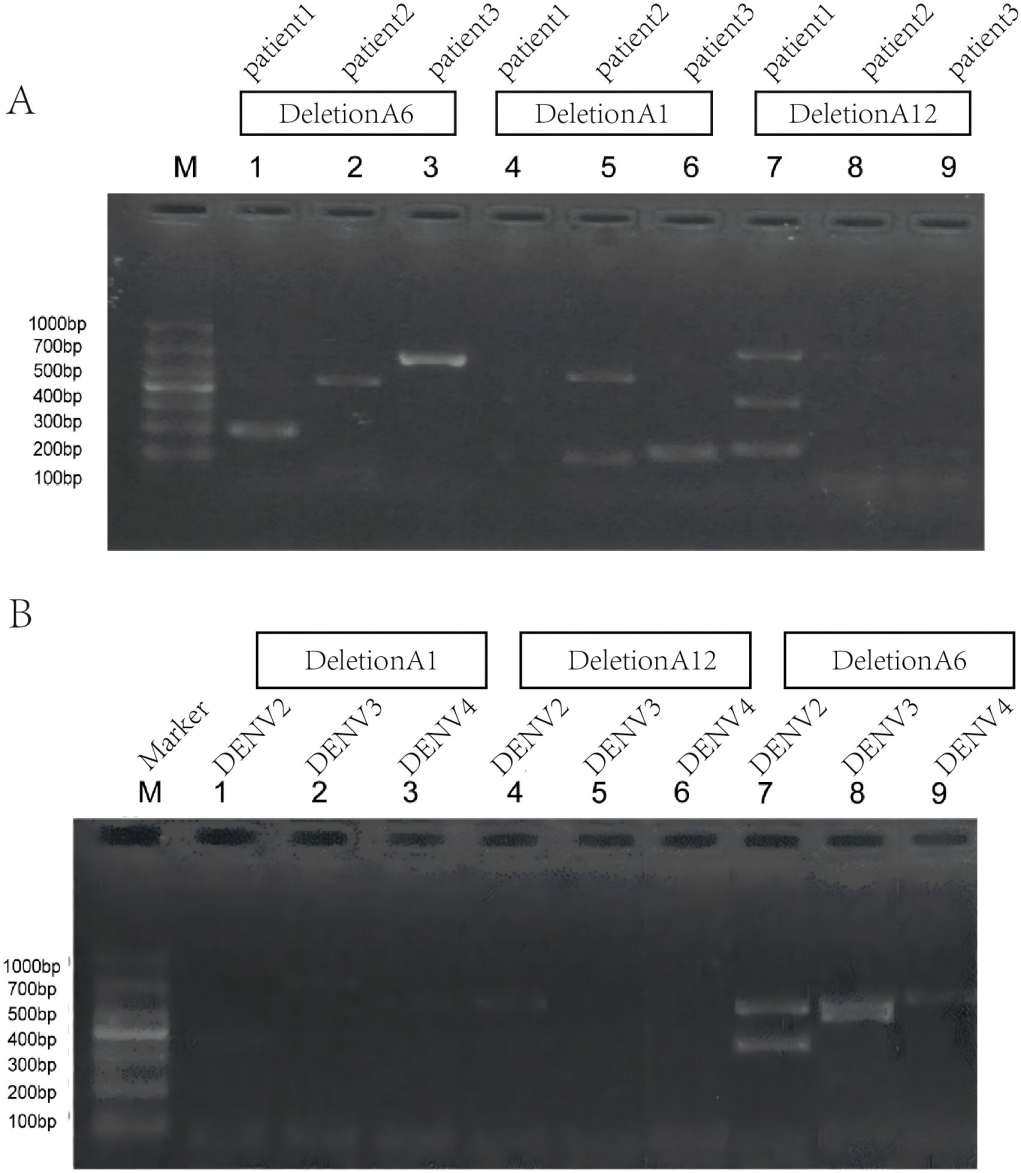
**(A)** Detection of Deletion A6 in sera from DENV1-infected patients. M: 1 kb DNA marker. Lanes 1–3, 4–6, and 7–9 correspond to PCR amplification of patient sera using primer sets specific for DeletionA6, DeletionA1, and DeletionA12, respectively. A band corresponding to the expected size of DeletionA6 was observed in lane 3. **(B)** Detection of Deletion A6 in sera from patients infected with DENV2, DENV3, and DENV4. M: 1 kb DNA marker. Lanes 1–3, 4–6, and 7–9 represent amplification using primer sets for DeletionA1, DeletionA12, and DeletionA6, respectively. Bands corresponding to the expected size of DeletionA6 were detected in lanes 7–9.

To further validate whether DeletionA1, DeletionA6, and DeletionA12 are real in DENV1–4 clinical samples, we first collected three DENV-1 clinical samples, extracted viral RNA from the sera, reverse transcribed the RNA, and performed PCR using primers specific for DeletionA1, DeletionA6, and DeletionA12. We found a PCR band matching the length of Deletion A6 in the serum of one patient (lane 2, **Figure 7A**). In order to verify that DeletionA6 is also present in patients with DENV-2, DENV-3, and DENV-4, we compared the sequences of the four DENV and found that the primer sequences used to detect DeletionA6 were conserved; therefore, we used the same primers for DeletionA6 for the assay and found that bands matching the length of DeletionA6 were present in sera from patients with DENV-2, DENV-3, and DENV-4 (**Figure 7B**). We then purified the DNA from the bands (lanes 7-9, **Figure 7B**) found in the patient’s serum that met our expectations, cloned them into vectors by TA cloning, and subsequently performed Sanger sequencing, which was compared with the genomes of DENV-1, DENV-2, DENV-3, and DENV-4, and revealed clear traces of gene jumps (**Supplementary Figure 4**). The BP as well as the RI of Deletion DVGs found in sera from DENV1–4 patients were very similar.

## 4 Discussion

In studies of DENV-derived defective viral genomes (DVGs), multiple short deletion-type DVGs were identified in the sera of patients during the acute phase of infection (Beauclair et al., 2018). Their capacity to form DIPs and suppress viral replication both *in vitro* and *in vivo* has been experimentally validated.

In a study of ZIKV DVGs, Rezelj et al. (2021) serially passaged Zika virus in various susceptible cell lines and employed NGS to detect and compare deletion-type DVGs across successive viral generations. They found that a cluster of deletion DVGs was consistently detected in multiple susceptible cell types, and their abundance increased with serial passaging. These deletion DVGs could associate with viral structural proteins to form DIPs, exhibiting self-replication ability and inhibitory effects against ZIKV both *in vitro* and *in vivo*.

NGS has become a common method in the study of different viral DVGs (Killip et al., 2013; Beauclair et al., 2018; Li et al., 2021; Zhou et al., 2022), which overcomes the limitations of traditional PCR methods to rapidly and comprehensively detect, classify, quantify, and characterize DVGs in different samples. Currently, the study of DENV DVGs, its focus is on Deletion DVGs (Li et al., 2011; Li and Aaskov, 2014; Timm et al., 2014), and the presence as well as the number of other types of DVGs is not yet known. Therefore, in our study, we use NGS combined with the DVGfinder software to analyze DENV DVG with the public DENV NGS databases, which included the NGS for DENV-3 acute-phase patients, NGS for Vero and C6/36 passaged by DENV-1 and *Aedes albopictus* passaged by DENV-1.

*In vivo*, Copyback DVG along with Deletion DVG were detected in the sera of our patients. 90% of the Copyback DVG detected was 3’ Copyback DVG. We quantified these different types of DVG and found that Copyback DVG was greater in number than Deletion DVG. Similarly, Deletion DVGs as well as Copyback DVGs were detected in *Aedes albopictus* infected with DENV-1. 5’ Copyback and 3’ Copyback DVGs were detected, and the number of Deletion DVGs was only 10^2, whereas the number of Copyback DVGs reached 10^5; *in vitro*, the DVGs detected in DENV-1-infected C6/36 and Vero were consistent with those detected in *Aedes albopictus*. Therefore, our data suggest that DENV-1 generates abundant copyback DVGs both *in vivo* and *in vitro*. This observation appears to be independent of host species.

In this study, our bioinformatic analysis revealed the presence of abundant copyback DVGs in DENV-1, a finding not previously described in NGS-based studies to our knowledge. However, we acknowledge that this observation is currently supported solely by computational evidence and requires orthogonal experimental validation (e.g., cloning or Northern blot) to be substantiated.

The replication mechanism of Deletion DVG is the same as that of normal viruses, which requires genomic 5’ UTR as well as 3’ UTR interactions to provide RdRp binding sites. We hypothesize the existence of a mechanism that requires only the 3’ UTR/5’ UTR and thus replication. Unlike Deletion DVG generation, Copyback DVG generation is where the RdRp is detached from the template and binds again to the nascent strand (Ziegler and Botten, 2020). Thus, Copyback has the viral genome 5’ UTR or 3’ UTR and the corresponding reverse complementary sequence. According to the definition of DVG, DVG is able to compete with normal genes to bind viral resources such as RdRp or structural proteins to inhibit viral replication (Olmo-Uceda et al., 2022). Similarly, this competitive relationship also exists between DVGs. In DENV-1, Copyback DVGs are much more abundant than Deletion DVGs, which may be due to the fact that: (1) Copyback DVG has a more efficient replication mechanism and binds RdRp efficiently, thus inhibiting the production of Deletion DVGs. (2) Due to the properties of the viral genome itself, it is more likely to produce large amounts of copyback DVG. (3) Both of the above possibilities exist.

Theoretically, DVGs can effectively activate innate immunity and thereby exert antiviral effects. In particular, copyback DVGs possess a stem-loop structure that efficiently stimulates innate immune pathways and are currently considered among the strongest known immune agonists. As mentioned above, approximately 90% of the copyback DVGs identified in patient sera were 3′ copyback species, suggesting that their enrichment *in vivo* may not be random. Similar preferential accumulation of copyback DVGs in clinical samples has also been reported during Sendai virus infection, where these molecules were shown to effectively activate innate immunity (Tapia et al., 2013). Therefore, the predominance of 3′ copyback DVGs observed in our study suggests that these molecules may participate in host antiviral responses and contribute to the clinical heterogeneity of Dengue virus infection. Investigating the correlation between copyback DVG abundance and patients’ innate immune status would be highly valuable. However, owing to the limited sequencing depth of NGS, further studies are still required to validate this hypothesis.

We attempted to find the stable presence of DENV DVGs by integrating them found in different datasets, which need to meet the following requirements: (1) they can be detected in different DENV-infected cell lines as well as *Aedes albopictus*, and are present in every generation during virus passage; (2) they can be detected in patient sera.

Different DVGs can adapt to different host environments. Therefore, on this basis, we defined the same DVGs that could be detected in each generation during the virus passage as host-adapted DVGs. The term “multihost-adapted” is used descriptively in this study to indicate consistent detectability across host-derived samples, and does not constitute evidence of cross-host transmission, packaging, or lineage continuity of the identified DVGs. Functional studies are required to determine whether these DVGs can be genuinely transmitted between hosts. We compared the production of Deletion DVGs by different DENV-1 (30A and 1806) during the passage in different hosts (C6/36, Vero, *Aedes albopictus*), and we found that a small fraction of the Deletion DVG could be consistently detected. Among these Deletion DVGs, some of them are extremely similar in BP and RI. In order to separate the dissimilar Deletion DVGs in a more reasonable way, we used the DBSCAN clustering algorithm to perform clustering analysis, and the clustering results were used as the host-adapted Deletion DVGs. After that, we comprehensively compared host-adapted deletion DVGs with those detected in patient sera and identified a subset present in both conditions. To further characterize these shared DVGs, we applied the DBSCAN clustering algorithm again to all host-adapted and patient-derived DVGs. Clusters containing DVGs from both sources were selected and defined as stably existing deletion DVGs.

Based on our bioinformatic analysis, we verified its accuracy by experimental methods. According to the principle of Deletion DVG generation, a part of the Deletion DVG sequence will be missing compared to the normal genome. Most of our predicted stabilized DVGs are greater than 2 kb in length, so it is difficult to amplify the full length. In order to improve the sensitivity of detection, we only amplify the sequences with traces of RdRp skipping, so we design 5’ primers upstream of the BP and 3’ primers downstream of the RI. The length of the predicted PCR-amplified fragments is less than 1 kb. According to the previous analysis, the number of Deletion DVGs was small. To improve the detection rate, we designed the nested PCR assay. Then, we cloned the PCR fragments into the vector, followed by Sanger sequencing, and compared the PCR sequences with normal viral sequences to identify Deletion DVG and its BP and RI by finding the jumping genes. We successfully cloned DNA bands of the expected length of DeletionA6 in sera of DENV-1 patients as well as in C6/36 and Vero-derived DENV-1, and we found that there were traces of gene jumps in these sequencing results and that the BP and RI were similar to those predicted by our characteristics of DeletionA6. Therefore, we confirmed that DeletionA6 is present in C6/36 and Vero cells infected with DENV-1, as well as in patient sera. Notably, of the 12 bioinformatically predicted candidates, only DeletionA6 remained positive after two rounds of nested PCR screening; the other 11 were not detected under our experimental conditions. While these negative results do not conclusively rule out their existence, further validation using alternative approaches is needed.

Dengue virus has four serotypes with 65%-70% sequence similarity among them (Zhao et al., 2021). We first found the position of DeletionA6 BP and RI on the viral genome, then we compared the complete genomes of DENV1–4 and found that the sequence of DENV1–4 was conserved around DeletionA6 BP and RI. Therefore, we hypothesized that DENV2–4 could also generate DeletionA6. We therefore performed nested PCR to detect DeletionA6 in the sera of DENV2–4 patients. Bands of the expected size were amplified, followed by sequencing and genome alignment analysis. Ultimately, we successfully cloned deletion-type DVGs fragments containing viral gene rearrangements from the sera of DENV2–4 patients. Comparative genomic analysis revealed that the breakpoint (BP) and reinitiation (RI) sites of these deletion DVGs were highly similar, although not completely identical. We considered that they belonged to the same class of Deletion DVGs as DENV-1 Deletion A6, sharing similar structural characteristics, with the BP located on the E gene and the RI site on the NS5 gene.

Deletion DVGs arise when the viral replicase is detached from the template and subsequently recombines to the same template. The reason for the detachment and recombination of the replicase is not clear, but it has been suggested that it is related to the sequence of the genes and secondary structure of the templates. Studies of other viral DVGs (Kolakofsky, 1979; Van Slyke et al., 2015; Kupke et al., 2019)have shown that the production of DVGs is not random and is regulated by genomic sequences, and further studies are needed to determine whether DENV-1 is regulated in the same way. We compared the BP site of DENV1–4 DeletionA6 and found that GAAUA is more likely to be present before the BP site; we compared the RI site of DENV1–4 DeletionA6 and did not find any obvious similar sequences. The close relationship between DVG formation and RdRp has been demonstrated that specific gene sequences can effectively induce the shedding and re-binding of RdRp to the same template. It has been shown that specific gene sequences can effectively induce RdRp to shed and recombine to form DVGs. It has also been hypothesized that the secondary structure formed by the viral genome can effectively bring BP and RI closer together, thus facilitating RdRp to shed from BP and then transfer to the RI site. We acknowledge that the analysis of the GAAUA motif near the breakpoint sites is descriptive and lacks formal statistical support. To further demonstrate that DVG production by DENV is not random, we will focus on the effect of the GAAUA sequence on DVG production in future subsequent studies.

## 5 Conclusion

In this study, we analyzed DENV-1-related high-throughput sequencing data from public databases and present bioinformatic evidence suggesting that DENV-1 can produce abundant copyback DVGs *in vivo* and *in vitro*. We found that serial passaging of DENV-1 revealed host-dependent dynamics of DVG composition, with relatively stable DVG proportions in mosquito hosts but a marked accumulation of specific deletion DVGs in Vero cells. In addition, we bioinformatically predicted 12 Deletion DVGs that can exist stably across hosts by integrating the information of DENV DVGs from many different types of datasets. Among these, DeletionA6 was experimentally confirmed to be real. We further demonstrated that DeletionA6 is present in the sera of patients infected with DENV1–4 and initially explored the mechanism of DeletionA6 production and found that the sequences near the BP of the four DENV-produced DeletionA6s have similar GAAUA sequences, which may be related to the GAAUA sequences that may be associated with DeletionA6 production. The shortcomings of this study are (1) DENV-1-related NGS data included in this study were short reads. (2) This study focused only on DVG, and although it verified that DeletionA6 actually existed, it failed to further verify its ability to form DIP and its inhibitory effect on viral replication. (3) We failed to successfully clone copyback DVGs from DENV-1 and correlate them with the innate immune profile of the patient. (4) We initially identified GAAUA sequences that may be associated with DeletionA6 formation and need to further validate their role. (5) Given the limited number of clinical samples available, the observed predominance of 3’ copyback DVGs and the detection of Deletion A6 across serotypes should be interpreted with caution and are considered preliminary findings that require validation in larger, well-characterized clinical cohorts. (6) Negative controls were routinely included in all nested PCR assays and consistently showed no bands. However, due to batch-wise experimental procedures, control lanes were not included in the final compiled gel images presented in the main figures. A representative gel showing negative control results is provided as **Supplementary Figure 5**.

## Data Availability

The datasets presented in this study can be found in online repositories. The names of the repository/repositories and accession number(s) can be found in the article/**Supplementary Material**.
