# Supplementary material for: Bioinformatics identification of copyback and multihost-adapted defective viral genomes in dengue virus
Source: Front Cell Infect Microbiol. 2026 May 20;16:1825608. doi: 10.3389/fcimb.2026.1825608 (PMC13229894; doi:10.3389/fcimb.2026.1825608)
Supplement: Supplementary file 1 [file Table1.docx]

Supplementary Material

# Supplementary Figures and Tables

## Supplementary Table

**Table. S1** NGS data used in this study

| Bioproject | DENV genome numbering (NCBI) | Descriptions | Host |
| --- | --- | --- | --- |
| RRJNA449278 | GenBank: HG316482.1  GenBank: MG518567.1 | DENV-1 30A, 10 times passage in C6/36 and *Aedes albopictus*; DENV-1 1806, 10 times passage in C6/36 and *Aedes albopictus* | C6/36 and Aedes albopictus |
| RPJNA669806 | GenBank: MW362471.1 | DEVN-1, Thailand 2005, 5 times passage in Vero | Vero |
| PRJNA895688 | GenBank: ON103303.1 | 24 patient samples | human |

**Table. S2** DBSCAN clustering parameters (eps and minPts) used for each dataset.

| Smple | eps | minpts |
| --- | --- | --- |
| 30A_C6/36 | 500 | 5 |
| 30A_Mosquito | 500 | 5 |
| 1806_C6/36 | 500 | 5 |
| 1806_Mosquito | 200 | 10 |
| 30A_Vero | 200 | 5 |
| Patient | 500 | 3 |
| Combined | 200 | 6 |

## Supplementary Figures


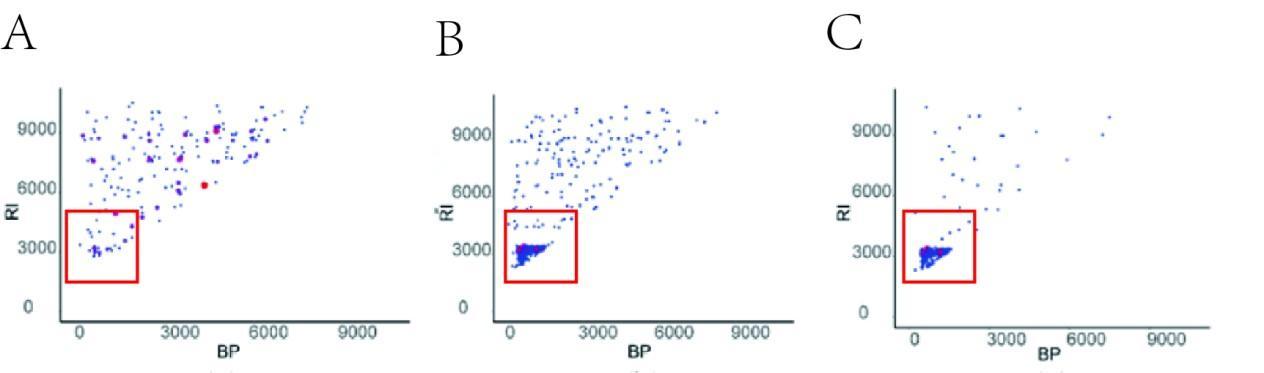


**Figure S1.** Specific Deletion DVGs accumulate during DENV-1 passage in Vero. Shown inside the red box is this particular class of Deletion DVGs. (a) Distribution of Deletion DVGs during the first passage of DENV-1 in Vero. (b) Distribution of Deletion DVGs during the third passage of DENV-1 in Vero. (c) Distribution of Deletion DVGs during the fifth passage of DENV-1 in Vero. This particular class of DVGs has BP of 0-1000 and RI of 2000-3000. As the virus was passaged, more DVGs appeared in the red box.

**
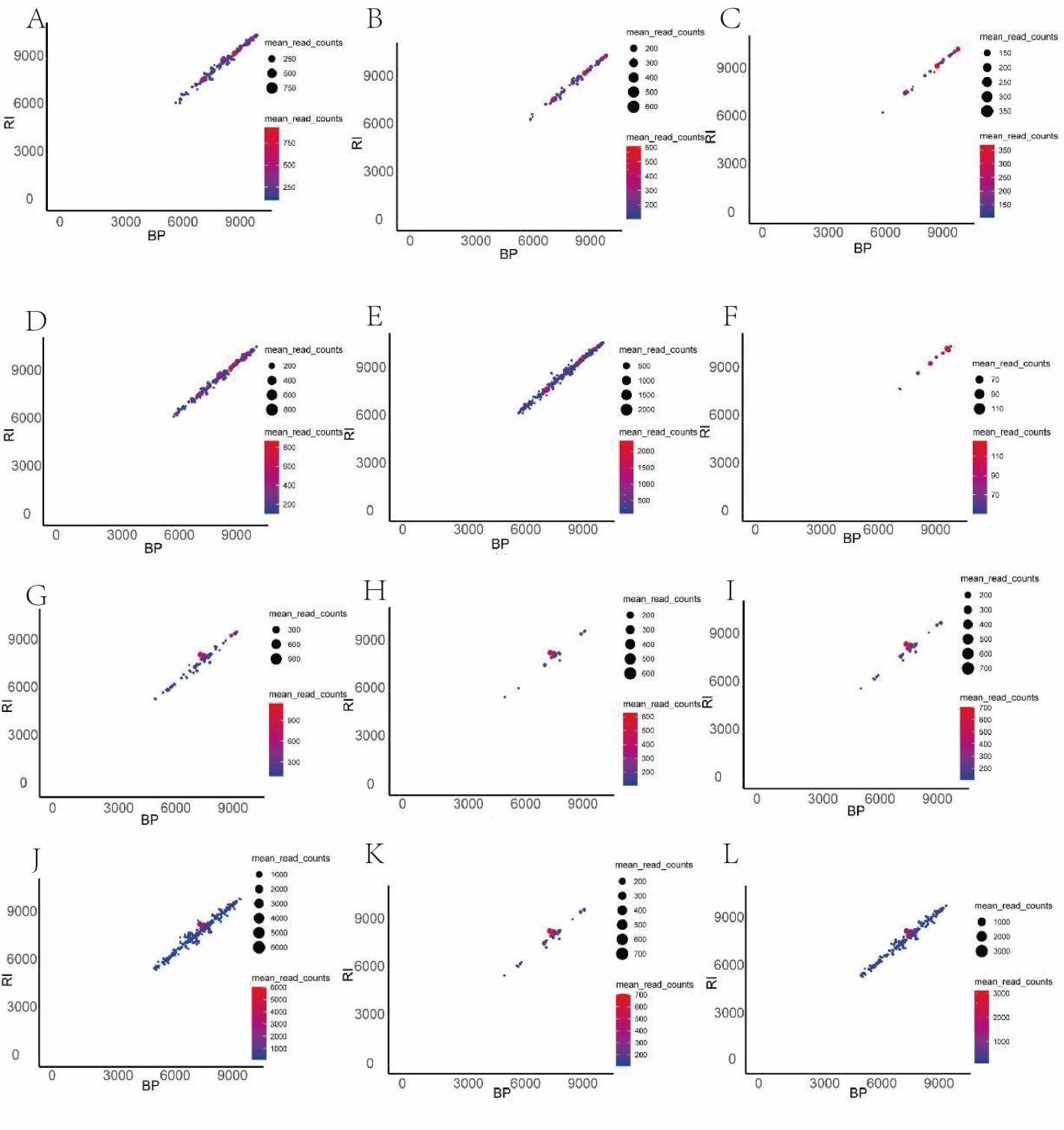
**

**Figure S2.** 3' copyback DVGs produced by DENV-1 in different hosts. We used bubble plots to describe the 3' copyback DVGs, with the horizontal coordinate indicating the position of the BP, the vertical coordinate indicating the position of the RI, and the size of the bubble indicating the reads of DVGs. (a-c) 3' copyback DVGs produced by DENV-1 30A in *Aedes albopictus* in first, fifth, and tenth passages. (d-f) 3' copyback DVGs produced by DENV-1 30A in C6/36 in first, fifth, and tenth passages. (g-i) 3' copyback DVGs produced by DENV-1 1806 in *Aedes albopictus* in first, fifth, and tenth passages. (j-l) 3' copyback DVGs produced by DENV-1 1806 in C6/36 in first, fifth, and tenth passages.

**
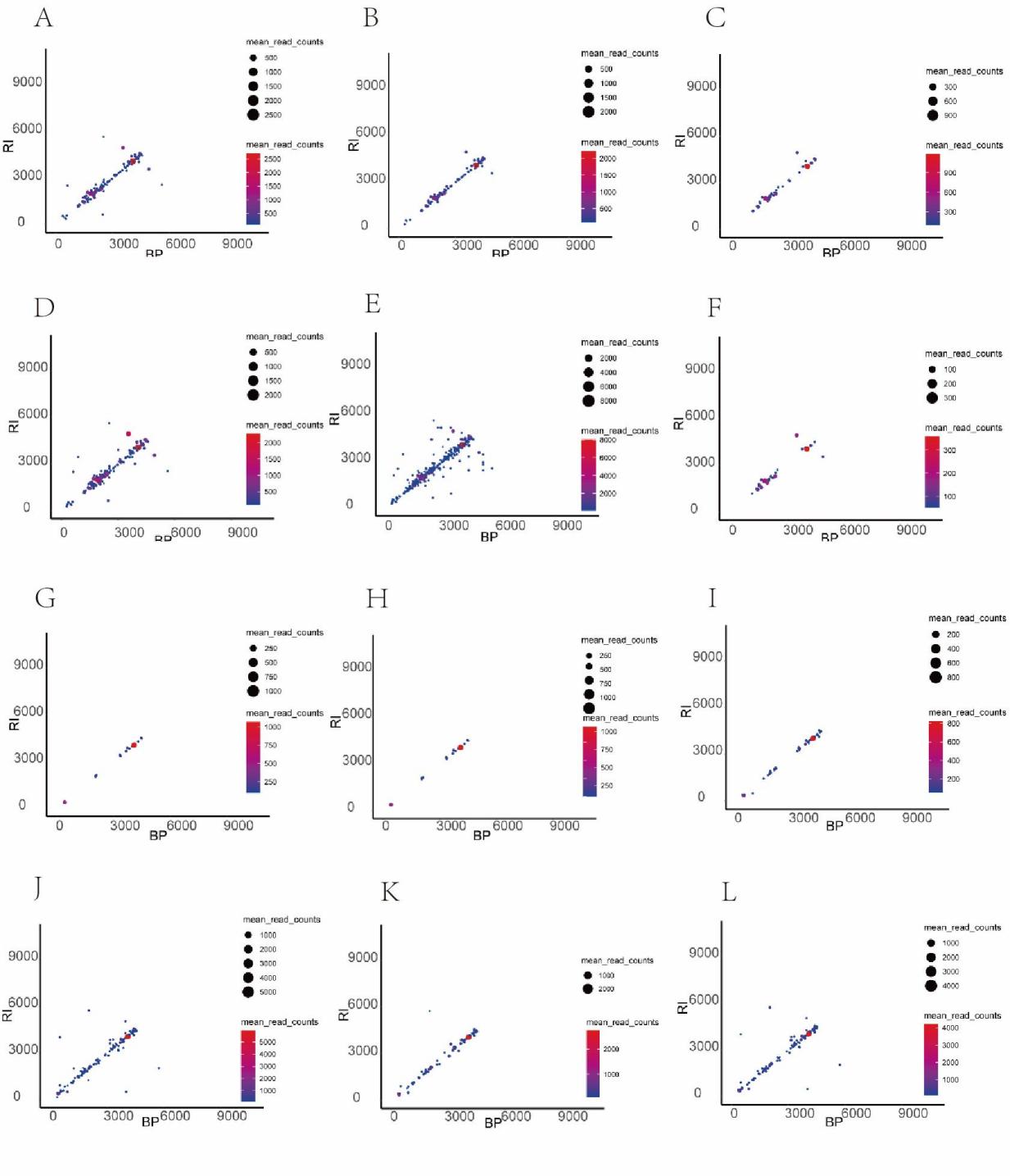
**

**Figure S3.** 5' copyback DVGs produced by DENV-1 in different hosts. We used bubble plots to describe the 5' copyback DVGs, with the horizontal coordinate indicating the position of the BP, the vertical coordinate indicating the position of the RI, and the size of the bubble indicating the reads of DVGs. (a-c) 5' copyback DVGs produced by DENV-1 30A in *Aedes albopictus* in first, fifth, and tenth passages. (d-f) 5' copyback DVGs produced by DENV-1 30A in C6/36 in first, fifth, and tenth passages. (g-i) 5' copyback DVGs produced by DENV-1 1806 in *Aedes albopictus* in first, fifth, and tenth passages. (j-l) 5' copyback DVGs produced by DENV-1 1806 in C6/36 in first, fifth, and tenth passages.

**
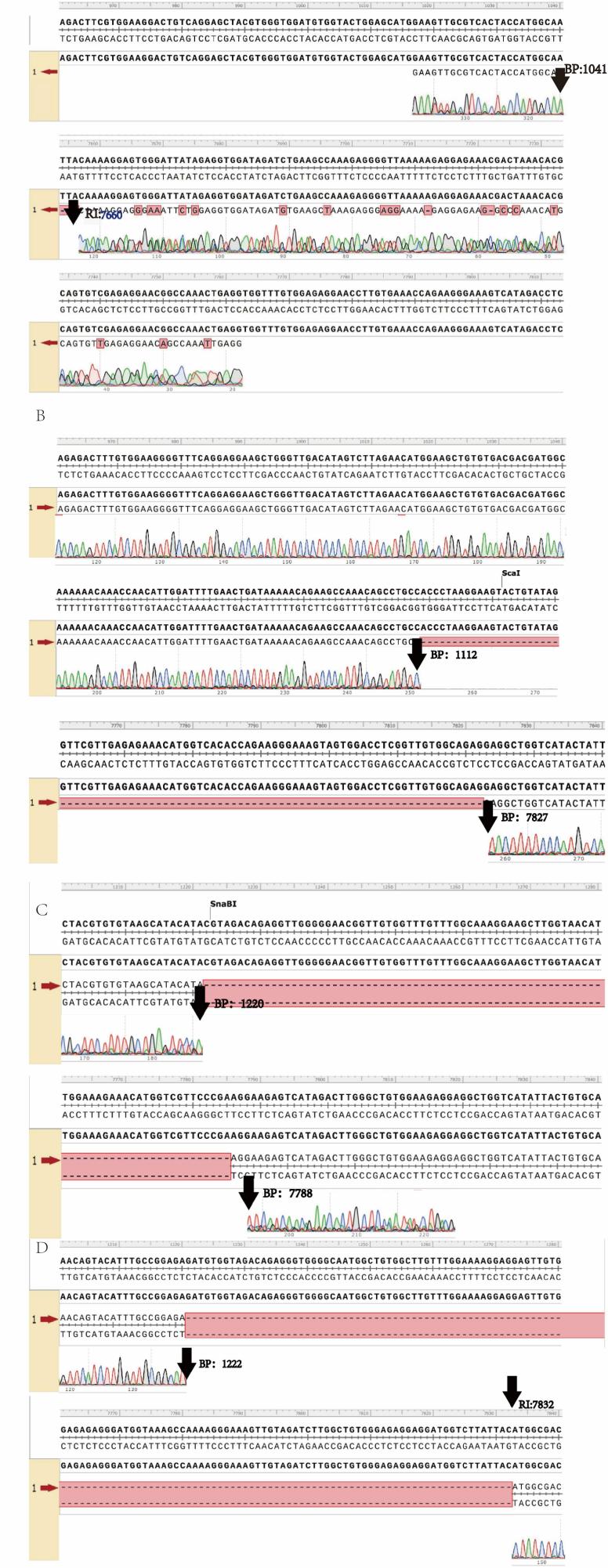
**

**Figure S4.** Sanger sequencing results of Deletion DVGs in serum from DENV1-4 patients. We amplified the corresponding Deletion DVGs in the sera of DENV1-4 patients using primers targeting DeletionA6, purified the DNA, cloned it into vectors using TA cloning, determined the bases using Sanger sequencing, and aligned the sequencing results to the complete genomes of DENV1-4. (a) We found the presence of Deletion DVGs with a BP of 1041 and an RI of 7660 in the sera of DENV-1 patients. (b) We found Deletion DVGs with a BP of 1112 and an RI of 7827 in the serum of DENV-2 patients. (c) We found Deletion DVGs with a BP of 1220 and an RI of 7788 in the serum of DENV-3 patients. (d) We found Deletion DVGs with a BP of 1222 and an RI of 7832 in sera of patients with DENV-4.


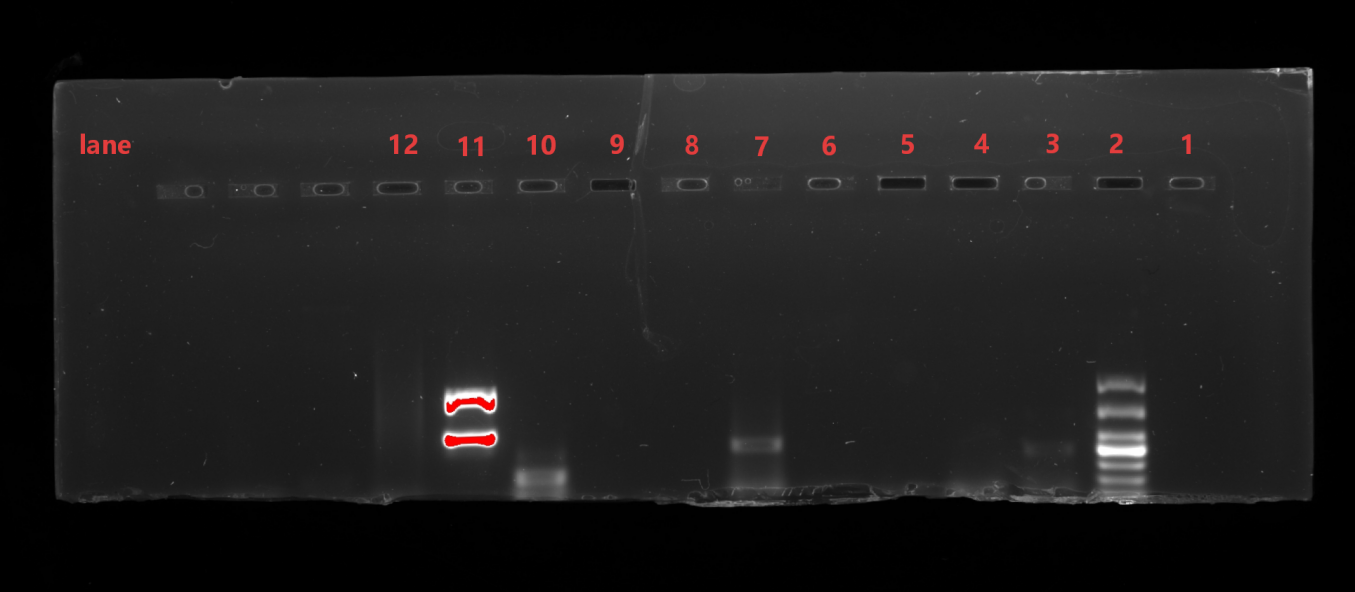


**Figure S5.** Representative nested PCR gel including negative controls. Lane order (right to left): Lane 1: no-template control (NTC); Lane 2: DNA marker; Lanes 3-11: sample amplicons; Lane 12: uninfected cell extraction blank. Both negative control lanes (NTC and uninfected cell extraction blank) show no visible bands, demonstrating the absence of contamination in this representative assay.

# Supplementary Methods

## DBSCAN clustering

Density-Based Spatial Clustering of Applications with Noise (DBSCAN), proposed by Martin Ester et al., is a density-based clustering algorithm that identifies clusters as regions of high point density separated by sparse regions. It does not require a predefined number of clusters and is capable of detecting arbitrarily shaped clusters while explicitly accounting for noise.

The method is governed by two parameters: the neighborhood radius (ε) and the minimum number of points (MinPts). Points are classified as core, border, or noise based on local point density within the ε-neighborhood. Clusters are defined as maximal sets of density-connected points and are iteratively expanded from core points by aggregating all density-reachable neighbors.

Detailed algorithmic procedures of DBSCAN are described in the original publication by Martin Ester et al. (1996), which corresponds to reference 19 in the main text..
